# Artificial Intelligence in Clinical Decision-Making: Current Applications, Challenges, and Future Directions in Modern Healthcare

**DOI:** 10.7759/cureus.107847

**Published:** 2026-04-27

**Authors:** Aditya Swaprakash Gadepalli Sri Pratyak

**Affiliations:** 1 AI and Healthcare, Global Alliant Inc, Clarksburg, USA

**Keywords:** algorithmic bias, artificial intelligence, clinical decision-making, deep learning, healthcare technology, machine learning, precision medicine

## Abstract

Artificial intelligence (AI) has emerged as a major driver of transformation in clinical decision-making and healthcare delivery systems. Machine learning, deep learning, natural language processing, and computer vision are increasingly being integrated into clinical workflows to support diagnosis, risk prediction, treatment planning, and operational efficiency. This narrative review synthesizes recent literature on the role of AI in clinical decision-making across key domains, including medical imaging, electronic health record analysis, precision medicine, clinical risk stratification, surgical support, and drug discovery. It also examines major barriers to safe and effective implementation, particularly algorithmic bias, limited external validation, data privacy concerns, poor interpretability, workflow disruption, and regulatory uncertainty. Ethical and medicolegal issues, including transparency, accountability, equity, and the effect of AI on clinician-patient relationships, are also discussed. Current evidence suggests that AI performs well in selected narrow tasks, especially in image-based and prediction-focused applications, but its reliability and clinical value remain inconsistent in complex real-world settings. Future progress is likely to depend on stronger prospective validation, explainable and multimodal systems, privacy-preserving learning approaches, and better integration of AI into clinical practice. The responsible use of AI in healthcare will require a multidisciplinary, patient-centered approach that balances innovation with safety, ethics, and clinical usefulness.

## Introduction and background

Healthcare is undergoing rapid digital transformation, driven by the expansion of biomedical data, advances in computing power, and the growing maturity of artificial intelligence (AI) methods. Clinical decision-making, traditionally guided by clinician expertise, clinical guidelines, and evidence-based medicine, is increasingly being supported by AI-enabled systems that can analyze large and complex datasets more efficiently than conventional approaches [1]. This shift is particularly relevant in modern healthcare, where the volume of medical knowledge and the complexity of diagnostic and therapeutic options continue to grow [2].

AI is an umbrella term that includes machine learning (ML), deep learning, natural language processing (NLP), computer vision, and related computational methods. In medicine, these technologies are being applied to a wide range of tasks, including radiological interpretation, pathology, genomic analysis, risk prediction, real-time monitoring, and clinical workflow support [3]. Their adoption has accelerated because of the availability of large clinical datasets, improvements in algorithmic design, and growing regulatory and commercial interest. More than 500 AI-enabled medical devices have received authorization from the US Food and Drug Administration, underscoring the expanding role of AI in clinical care [4].

Despite this momentum, the use of AI in clinical decision-making remains contested. Concerns persist regarding algorithmic opacity, bias, lack of generalizability, privacy risks, workflow disruption, and incomplete regulatory oversight [5]. These concerns are not theoretical: several deployed systems have shown reduced performance, bias, or poor transferability when applied in real-world clinical environments outside their development settings [6]. Accordingly, enthusiasm for AI must be balanced with careful evaluation of its safety, validity, and practical usefulness.

This narrative review was undertaken to synthesize the recent literature on AI in clinical decision-making and to provide an integrated overview of its current applications, major limitations, and future directions. Specifically, this review aims to (1) summarize current AI applications across major clinical domains; (2) critically examine barriers to implementation, including technical, ethical, and regulatory challenges; and (3) identify priority directions for responsible and clinically meaningful adoption of AI in practice.

## Review

Foundational concepts: AI technologies in healthcare

ML and Deep Learning Paradigms

ML forms the basis of most clinical AI applications by enabling algorithms to learn patterns from data and use them for prediction, classification, or decision support without explicit rule-based programming. Supervised learning methods such as support vector machines, random forests, logistic regression, and gradient boosting have been widely applied in clinical classification and regression tasks, including the prediction of mortality, hospital readmission, disease progression, and treatment response [7]. Unsupervised methods, including clustering and dimensionality reduction, are mainly used to identify hidden patient subgroups and explore complex high-dimensional datasets. Reinforcement learning has also shown promise for sequential clinical decisions, such as medication dosing, ventilator control, and treatment sequencing in oncology [7].

Deep learning, a subset of ML based on multilayer neural networks, has demonstrated strong performance in unstructured clinical data, especially images, free-text notes, and physiological signals [8]. Convolutional neural networks are widely used in medical image analysis, while recurrent and transformer-based models are increasingly applied to longitudinal clinical records, electrocardiograms, and electroencephalograms [9]. However, although these methods can achieve high technical performance, their complexity contributes to concerns about interpretability, generalizability, and safe clinical translation.

NLP in Clinical Documentation

NLP enables the extraction of clinically meaningful information from unstructured sources such as electronic health records (EHRs), radiology reports, pathology reports, discharge summaries, and biomedical literature. This is particularly important because a large proportion of clinical information remains text-based and cannot be directly analyzed using traditional structured-data approaches. NLP applications in healthcare include named entity recognition, relation extraction, text classification, summarization, and clinical information retrieval.

Domain-specific language models trained on biomedical corpora have shown increasing usefulness in summarizing patient histories, identifying diagnostic clues, and supporting clinical documentation [10]. Large language models have also demonstrated strong performance on medical knowledge tasks, including licensing-style examinations [11]. Nevertheless, their clinical use remains constrained by hallucination, contextual inconsistency, and uncertain reliability in high-stakes settings. As a result, current NLP systems are best viewed as assistive tools rather than autonomous clinical decision-makers.

Signal Processing and Computer Vision

Computer vision is one of the most mature areas of clinical AI, particularly in medical image interpretation. Deep learning models have shown strong performance in detecting lesions, identifying anatomy, quantifying disease burden, and assessing treatment response across radiology, pathology, dermatology, and ophthalmology. Beyond static images, computer vision has also been explored for video-based tasks such as surgical phase detection, endoscopic lesion identification, rehabilitation assessment, and fall-risk prediction.

AI-based signal processing has similarly shown value in continuous physiological monitoring, including electrocardiographic, electroencephalographic, and hemodynamic data. Deep learning models have demonstrated the ability to identify arrhythmias, seizure onset, and hemodynamic instability with clinically meaningful lead times, potentially enabling earlier intervention and better patient outcomes [9]. However, real-world usefulness depends not only on predictive accuracy but also on signal quality, prospective validation, and effective integration into bedside workflows.

Current clinical applications of AI in decision-making

Medical Imaging and Diagnostic Radiology

Medical imaging remains the most established domain of clinical AI because of the availability of large labeled datasets and the visually structured nature of many diagnostic problems. Deep learning models have shown high performance across selected imaging tasks and, in some settings, have matched or exceeded expert radiologists.

In breast cancer screening, a deep learning system improved performance in validation cohorts from the United States and the United Kingdom, reducing false-positive and false-negative rates [12]. Autonomous diabetic retinopathy screening systems have also demonstrated high sensitivity and have enabled specialist-independent screening in primary care environments [13]. Similar gains have been reported in chest radiography and low-dose CT-based lung cancer screening, where AI may support earlier detection and risk stratification [14,15].

Despite these promising findings, much of the imaging literature remains methodologically limited. Many studies rely on retrospective designs, single-center cohorts, and insufficient external validation, leaving performance vulnerable to dataset shift across populations, scanners, and imaging protocols [16]. Thus, imaging is a relatively strong area for AI, but even here, evidence of reliable real-world benefit remains incomplete.

Clinical Risk Prediction and Early Warning Systems

AI-based risk prediction models have been developed for sepsis, acute kidney injury, cardiac arrest, hospital readmission, and general clinical deterioration. These systems use structured EHR data to identify subtle patterns in laboratory values, vital signs, and clinical trajectories that may precede adverse outcomes.

Sepsis prediction illustrates both the promise and the limitations of this area. Although commercial sepsis models have been widely deployed, external validation of a widely used proprietary system found substantially worse performance than originally reported, raising concerns about transparency, reproducibility, and generalizability [17]. In contrast, some newer models for acute kidney injury have shown encouraging performance in more rigorous evaluation settings, including prediction of severe kidney injury before overt clinical presentation [18].

Overall, early warning systems are clinically attractive, but their success depends on more than algorithmic performance. Alert timing, actionability, workflow integration, and avoidance of alert fatigue are critical to determining whether they improve care or simply add noise.

Precision Medicine and Genomics

AI has become increasingly important in precision medicine because genomic and molecular datasets are too large and complex for conventional interpretation alone. Deep learning approaches have improved variant calling from next-generation sequencing data and are also being used for variant interpretation and pathogenicity prediction [19].

In oncology, AI models have been trained to infer molecular features and treatment response from integrated tumor and histopathology data [20]. These methods may reduce dependence on expensive molecular testing in selected settings, although broader validation is still needed. AI has also been applied to pharmacogenomics, where models can support individualized drug dosing and the prediction of adverse reactions. For example, ML-based warfarin dosing has shown better performance than standard clinical dosing algorithms in some studies [21]. In addition, AI has been used to predict drug-drug interactions, support pharmacovigilance, and identify patients at higher risk of medication-related complications.

Drug discovery is another rapidly expanding area, with AI being used in target identification, lead optimization, and clinical trial prediction. However, compared with diagnostic imaging and risk prediction, the clinical impact of these applications remains less mature and more anticipatory than established.

Surgical Decision Support and Robotics

In surgery, AI is being explored across the perioperative pathway, including preoperative risk assessment, intraoperative guidance, postoperative complication prediction, and surgical training. Computer vision analysis of surgical video has shown the ability to identify procedural phases, anatomical landmarks, adverse events, and technical skill, with potential applications in quality assurance and intraoperative support [22].

AI-enhanced robotic systems may improve technical precision and support navigation or tissue recognition, but current evidence for improved patient outcomes over conventional approaches remains inconsistent [23]. Preoperative models may help predict complications, length of stay, and mortality, while postoperative tools are being investigated for wound assessment, infection prediction, and early complication detection.

Overall, surgical AI remains promising but is relatively early in clinical maturity. Much of the literature is developmental, and stronger prospective validation is needed before broad claims of outcome improvement can be justified.

Mental Health and Behavioral Applications

AI applications in mental health include the prediction of suicide risk, detection of depressive symptoms, identification of psychosis-related risk, and personalization of psychotherapeutic interventions. NLP has been used to identify undocumented depression and anxiety in clinical notes, while digital phenotyping approaches use passive smartphone and sensor data to infer behavioral and psychological states.

These applications may help address gaps in access to mental health care, but they also raise unusually strong concerns regarding privacy, surveillance, consent, and interpretive validity [5]. Accordingly, this area illustrates both the potential reach of AI and the ethical sensitivity of deploying such systems in vulnerable populations.

Challenges and barriers to clinical AI implementation

Algorithmic Bias and Health Equity

One of the most serious barriers to clinical AI is the risk of reproducing or amplifying existing healthcare disparities. Because AI systems are trained on historical data, they may learn patterns shaped by unequal access to care, differential treatment, and structural discrimination. If these biases are embedded in training data, AI models can propagate them at scale.

A widely cited study showed that a commercial algorithm used to predict healthcare needs underestimated illness burden in Black patients relative to White patients with similar disease severity, largely because it used healthcare costs as a proxy for need [24]. This example illustrates how apparently neutral design choices can encode structural inequities. Addressing such bias requires representative datasets, fairness-aware model design, subgroup performance auditing, and ongoing postdeployment monitoring [25]. It also requires broader representation across age, sex, gender, socioeconomic status, geography, and disability, as systems trained in highly resourced academic settings may not generalize to underserved populations or low-resource contexts.

Interpretability and Explainability

The limited interpretability of many deep learning systems is a major obstacle to clinical adoption. Clinicians are expected to justify decisions, communicate reasoning to patients, and detect errors in recommendations, all of which are harder when models function as black boxes [26].

Explainable AI approaches such as attention mapping, SHapley Additive exPlanations (SHAP) values, Local Interpretable Model Agnostic Explanation (LIME), and concept-based explanations have been developed to offer post hoc insight into model behavior [27]. However, these methods remain imperfect, and their clinical usefulness is still debated because explanations may not fully reflect the true decision process of the model. This creates a persistent trade-off: more complex models may improve predictive performance, but simpler or inherently interpretable models may be preferable in higher-risk clinical contexts.

Data Quality, Privacy, and Interoperability

Clinical AI systems depend heavily on the quality, completeness, and representativeness of training data. EHR data are often incomplete, inconsistently coded, and affected by documentation variability, which can impair model reliability and introduce spurious relationships [28]. Data preprocessing, cleaning, and variable standardization are therefore central rather than secondary stages of model development.

Privacy regulation also constrains data sharing and multi-institutional AI research. Although these protections are necessary, they make it harder to assemble diverse training datasets. Federated learning has emerged as a promising response, allowing models to be trained across institutions without centralizing patient data [29]. Interoperability remains another major challenge. Adoption of standards such as Fast Healthcare Interoperability Resources may support more scalable integration of AI into clinical workflows, but incomplete uptake and legacy systems continue to limit progress [30].

Regulatory and Medicolegal Considerations

Regulatory frameworks for clinical AI are still evolving and are not fully adapted to continuously learning systems whose performance may change after deployment [4]. Traditional premarket approval processes were designed for static devices and are less suited to adaptive AI models.

Medicolegal liability is also unresolved. When AI-related harm occurs, responsibility may be difficult to assign among clinicians, developers, and healthcare institutions [31]. Likewise, when clinicians disregard valid AI advice, questions may arise whether such action departs from the standard of care. These uncertainties remain a major barrier to confident implementation.

Clinician Trust, Workflow Integration, and Human Factors

Successful implementation of clinical AI depends not only on technical accuracy but also on acceptance by clinicians and integration into real-world workflows. Trust is shaped by transparency, agreement with clinical judgment, prior experience, and the perceived credibility of the system and its developer [32].

Human factors are central. Automation bias may lead clinicians to over-rely on AI recommendations, whereas automation disuse may cause them to ignore useful advice [33]. Alert fatigue further complicates implementation, especially when systems generate frequent low-value warnings. As a result, the real effectiveness of clinical AI depends on interface design, timing, actionability, and alignment with clinical routines.

Ethical dimensions of AI in clinical decision-making

The use of AI in clinical decision-making raises ethical concerns that extend beyond technical performance. These include patient autonomy, informed consent, accountability, distributive justice, and preservation of the therapeutic relationship. Patients may reasonably wish to know whether AI has influenced decisions about their care, yet practical standards for disclosure and consent remain underdeveloped [34].

There is also concern that growing reliance on algorithmic systems could depersonalize care and weaken empathy, trust, and narrative understanding in clinical encounters [35]. At the same time, proprietary systems may limit independent scrutiny and accountability, complicating ethical oversight [36]. These issues are especially important in light of distributive justice, as AI systems developed primarily in well-resourced settings may worsen rather than reduce health inequities if access and validation remain uneven.

Future directions

Multimodal AI and Digital Twins

A key future direction in clinical AI is multimodal integration, in which genomic, proteomic, metabolomic, imaging, wearable, environmental, and EHR data are combined within a single analytical framework. Such models have shown stronger predictive performance than unimodal approaches in areas such as oncology, cardiovascular disease, and neurodegenerative disorders [37]. By integrating multiple data streams, multimodal AI may provide a more complete representation of patient status and disease progression than any single source alone.

Patient-specific digital twins represent a related but less mature direction. These virtual models are intended to simulate disease trajectories and treatment responses using individualized patient data [38]. Although the concept is promising for personalized decision-making, current applications remain largely developmental, and their near-term clinical role is likely to be limited compared with more immediately feasible multimodal decision support systems.

Prospective Clinical Validation and Randomized Trials

Prospective validation is one of the most urgent needs in clinical AI. Much of the current literature remains retrospective, and relatively few systems have been tested in real-world clinical settings. Randomized controlled trials and other prospective designs are needed to determine whether AI-assisted decision-making improves patient outcomes, efficiency, or cost-effectiveness in practice [39].

Future studies should move beyond technical performance alone and assess workflow fit, clinician-AI interaction, and patient-centered outcomes. Trial frameworks such as CONSORT-AI and SPIRIT-AI are especially relevant because they provide clearer standards for reporting AI-focused clinical studies [39]. Strengthening prospective validation directly addresses the current gap between promising algorithmic performance and uncertain clinical benefit.

Education, Workforce Development, and Organizational Readiness

Effective adoption of AI will also depend on workforce and institutional readiness. Medical education should increasingly include AI literacy, critical appraisal of AI evidence, ethical awareness, and practical skills for human-AI collaboration [40]. These competencies are necessary because clinicians will be expected not only to use AI tools but also to judge their reliability and limitations in clinical practice.

At the institutional level, governance structures, monitoring systems, and implementation processes are equally important. Healthcare organizations must be able to evaluate deployed systems, respond to safety concerns, and ensure alignment with clinical workflows and ethical standards. These priorities are more immediately actionable than broader speculative future applications and are essential for translating AI from experimental promise into safe routine care.

Discussion

The evidence synthesized in this review indicates that AI performs best in narrow, technically defined clinical tasks, particularly medical image interpretation and pattern recognition from structured clinical data. Applications in imaging, risk prediction, precision medicine, surgical support, and mental health collectively demonstrate the breadth of AI’s potential in medicine. However, a consistent finding across domains is the gap between strong algorithmic performance and proven clinical impact. This gap reflects not only technical limitations but also regulatory, ethical, and organizational barriers that constrain translation into routine practice.

One of the most important observations is the mismatch between the volume of AI development studies and the relative scarcity of rigorous external and prospective validation. Many published models remain evaluated only in retrospective datasets, and even fewer have been tested in real-world clinical settings [16]. As a result, existing evidence often supports technical promise more strongly than practical clinical utility. Future progress will depend less on demonstrating incremental gains in benchmark accuracy and more on establishing reproducible evidence of safety, effectiveness, and cost-effectiveness in real care environments.

Algorithmic bias remains a central concern because it directly affects both fairness and patient safety. The literature shows that AI systems can reproduce structural inequities when training data reflect unequal access to care, differential treatment, or distorted proxy variables [24]. Addressing this problem requires more than diverse datasets alone; it also requires subgroup auditing, fairness-aware model development, and governance mechanisms that explicitly monitor equity-related performance after deployment [25]. In this respect, equity should be treated as a core performance criterion rather than a secondary ethical consideration.

Interpretability remains another unresolved challenge. Although more complex models may offer stronger predictive performance, their opacity can weaken clinician trust, complicate accountability, and reduce the ability to identify erroneous outputs [26,27]. The relevant question is therefore not simply whether a model is accurate, but whether its outputs can be meaningfully integrated into clinical reasoning. Similarly, implementation science and human factors are crucial to success: even well-performing systems may fail if they are poorly aligned with workflows, generate excessive alerts, or encourage automation bias [32,33].

Taken together, the findings of this review suggest that the next phase of clinical AI should focus on robust validation, equity-sensitive evaluation, workflow integration, and governance rather than technical novelty alone. The literature supports cautious optimism: AI has clear value in selected domains, but its broader contribution to clinical decision-making will depend on whether future systems can demonstrate reliable, explainable, and context-sensitive benefit in actual care settings.

## Conclusions

AI is transformative and can be used to improve clinical decision-making throughout the entire range of healthcare delivery, including primary prevention and early diagnosis, as well as the optimization of treatment and prognostic prediction. The existing data show that AI systems are able to pursue amazing results in certain clinical functions, indicating the potential of more precise, more efficient, and individualized care of patients. Nonetheless, to achieve this potential, one has to negotiate a complicated terrain of technical, ethical, regulatory, and organizational barriers that cannot be solved by using technological innovation alone. Clinical expertise, data science, ethics, regulatory science, and implementation science should be incorporated into a multidisciplinary, patient-centered approach to ensure that clinical AI is useful in achieving the ultimate aims of medicine, namely, in improving patient outcomes, reducing suffering, and facilitating health equity. Wisdom, rigor, and ethical dedication in the development, validation, and implementation of these dynamic tools in the service of human health will decide the future of AI in clinical decision-making based on not just the sophistication of algorithms but also the wisdom of algorithms.
